# Impure Air

**Published:** 1859-08

**Authors:** 


### IMPURE AIR.

The men who worked in the Thames Tunnel "suffered severely" by emaciation, low fevers, and even death, from breathing the deleterious gas of the place; when, by the most critical chemical tests there was but one part of bad air to a hundred thousand. No wonder then, that the atmosphere of a celebrated hotel, several years ago, caused so much sickness and suffering and death, when it was proven that when the witness went into one of the privies under the same roof, the filth under the floor spirted up through the cracks, upon the ordinary pressure of the foot. This was one of the diseases, the very essence of which was filth; and would never have had a place or a name, had there not been a putrifying air, and water, and more solid matter, to have given rise to it. A handful of charcoal, ignited in a small close room, will destroy the sleeper's life before morning. An atmosphere containing only two parts of carbonic acid gas in a hundred of common air, killed a puppy in two minutes and a half; and a dog, which breathed an atmosphere containing only a quarter of one per cent. of the same gas, died in ten hours.

Dull indeed must be the intellect which is not convinced by these facts, of the absolute necessity to health, of making it a constant study to secure the breathing of a pure atmosphere during the hours of sleep and of quietude in the house during day light.

The fire place should be always kept as open as possible in a sleeping room, both winter and summer. In winter, because the carbonic acid gas which comes from the lungs during respiration, and so small a proportion of which, as just shown, is destructive of animal life, is made heavy by the cold, and seeks the floor, when a slight draft from the cracks of the doors and windows, carries it up the chimney.

The fireplace ought to be kept open in summer, because, while the colder air of the night without falls down the chimney by its greater weight, the rarified and impure air of the interior of the house is constantly rising and escaping. Thus it is, that in any chimney, where there is no fire, two currents of air, in opposite states, are constantly passing, one up the other down.

One of the best means of ventilating a sick chamber, where it may be considered not advisable to raise a window, is to open an inner door, and kindle a chip fire on the hearth for a few minutes in summer, or simply open the door, if it is fire-time of year.

